# Long-term care transitions during a global pandemic: Planning and decision-making of residents, care partners, and health professionals in Ontario, Canada

**DOI:** 10.1371/journal.pone.0295865

**Published:** 2023-12-15

**Authors:** Sarah Carbone, Whitney Berta, Susan Law, Kerry Kuluski

**Affiliations:** 1 Institute of Health Policy, Management and Evaluation, University of Toronto, Toronto, Ontario, Canada; 2 Institute for Better Health, Trillium Health Partners, Mississauga, Ontario, Canada; Universidad de Santiago de Chile Facultad de Ciencias Medicas, CHILE

## Abstract

The COVID-19 pandemic appears to have shifted the care trajectories of many residents and care partners in Ontario who considered leaving LTC to live in the community for a portion or the duration of the pandemic. This type of care transition–from LTC to home care–was highly uncommon prior to the pandemic, therefore we know relatively little about the planning and decision-making involved. The aim of this study was to describe who was involved in LTC to home care transitions in Ontario during the COVID-19 pandemic, to what extent, and the factors that guided their decision-making. A qualitative description study involving semi-structured interviews with 32 residents, care partners and health professionals was conducted. Transition decisions were largely made by care partners, with varied input from residents or health professionals. Stakeholders considered seven factors, previously identified in a scoping review, when making their transition decisions: (a) institutional priorities and requirements; (b) resources; (c) knowledge; (d) risk; (e) group structure and dynamic; (f) health and support needs; and (g) personality preferences and beliefs. Participants’ emotional responses to the pandemic also influenced the perceived need to pursue a care transition. The findings of this research provide insights towards the planning required to support LTC to home care transitions, and the many challenges that arise during decision-making.

## Introduction

The COVID-19 pandemic has had a disproportionate impact on long-term care (LTC) globally [1] including the institutional LTC sector in Ontario, Canada where many LTC residents and care partners considered aging elsewhere over the pandemic [2]. In Ontario, LTC is used to describe institutional spaces where adults live and receive a range of health and personal care services [3]. Specifically, LTC refers to congregate care facilities where individuals live in individual or shared rooms and receive help with most or all daily activities and have access to 24-hour nursing and personal care [4]. LTC may be run by not-for-profit or for-profit companies [4]. The government pays for personal and nursing care while the individual pays for accommodation charges and optional additional services [4]. In Ontario, there are over 76,000 residents living in LTC with another 40,000 waiting for a bed [5]. From June to December 2021, 18.4% of Ontarian LTC residents who contracted COVID-19 died [6]. The case fatality ratio has since decreased to 3.7% in December 2022 [6]. Throughout this paper, we describe institutional LTC as LTC.

For decades, Ontario’s LTC sector has faced significant issues, including staffing shortages and poor infrastructure [7, 8], which were exacerbated during the pandemic [9]. New public health measures like visitor restrictions that were implemented to control the spread of COVID-19 also served to reduce the quality of life of residents who were confined to their rooms for prolonged periods of time [10, 11] and some research suggests that these measures accelerated cognitive decline and death [12, 13]. As a result of these issues, many residents and care partners (e.g., family) considered moving out of LTC settings during the pandemic [14–16]. The Government of Ontario also responded to the crisis in LTC by amending LTC regulations to allow residents to move out for a period of up to 3 months and then return to the top of the LTC waitlist [17, 18]. This regulatory change created the opportunity for residents to live with their families in the community while still reserving a space in an institutional care setting–an option that was previously not permitted.

Little is known at the individual level about the planning and decision-making behind these care transitions out of LTC settings during the pandemic. A care transition is the process of moving a patient between different settings or providers [19, 20]. Often regarded as high-risk events, care transitions can threaten patient safety by disrupting continuity in care, contributing to gaps in communication, and leading to adverse outcomes [21–23]. This is particularly true for older populations who have heightened vulnerability due to their complex care needs [24, 25]. To date, most of the literature on care transitions has focused on discharges from hospitals or rehabilitation facilities to home or other care institutions. Studies have been conducted on a variety of populations and areas, including interventions and innovations to improve transitional care [26–28], patient and family experiences [29, 30], and best practices [31]. A small body of research has explored care transitions in LTC settings; however, its focus has been on admission to LTC [32, 33] or emergency discharges to hospital [34]. Although many types of care transitions share common challenges, including caregiver burnout and poor access to home care resources [35], transitions from LTC to the community were a relatively rare phenomenon until the start of the COVID-19 pandemic. Transitions like these are likely to continue in the future in response to other infectious disease outbreaks or in the advent of new aged care pathways. Thus, further research is needed to better understand the rationale and decision-making behind these transitions, and how they were shaped by the pandemic circumstances.

Care transitions in LTC settings have important implications for multiple stakeholder groups. In general, safe transitions require coordination and planning between the patient, their care partners and health professionals from each setting involved [36]. In the case of LTC to community transitions, the impacts on residents are substantial as the transition results in both a change to their living environment and their care providers. Care partners are impacted by the decision to move a resident out of LTC as their caregiving responsibilities are likely to increase and in many cases the resident might be moving into their personal homes. Finally, health professionals employed in LTC and home care sectors may also be responsible for transferring or assuming accountability for the resident as they move. Despite the relevance of these decisions to each of these stakeholder groups, prior research has shown that transition decisions are often not shared by representatives of these groups [37].

Previously, our team conducted a scoping review that found that transition decisions in institutional care settings are typically led by health professionals with varied and often limited involvement of residents and care partners [37]. Several common factors influence these groups’ transition decision-making in multiple care sectors, including: (a) institutional priorities and requirements; (b) resources; (c) knowledge; (d) risk; (e) group structure and dynamic; (f) health and support needs; and (g) personality, preferences and beliefs. It remains unknown whether these factors are considered in LTC to community transitions, and how they may have been impacted by the COVID-19 pandemic. The aim of this study was to describe residents’, care partners’ and health professionals’ roles in transition planning in Ontario’s LTC settings during the COVID-19 pandemic and the factors that guided their decision-making.

## Methods

This qualitative study explored care transition decision-making in Ontario’s LTC sector during the COVID-19 pandemic. Interviews were conducted with residents, care partners, and health professionals who considered, supported, and/or experienced a care transition out of a LTC setting in Ontario during the pandemic. The aim of the study was to understand each of these stakeholder groups’ involvement in transition planning and the factors that guided their decision-making. A qualitative description design was used [38], involving in-depth semi-structured interviews with representatives from stakeholder groups. All interviews were conducted virtually due to the many public health measures adopted in Ontario during the pandemic and were completed between December 3, 2021 and August 5, 2022 following Canada’s fourth wave of the pandemic. Several health organizations from across Ontario facilitated participant recruitment by sharing study information within their networks. Interviews were conducted by phone or videoconference (i.e., Zoom) depending on participant preferences. Written or verbal consent was obtained prior to the initiation of the interview. The University of Toronto’s Health Sciences Research Ethics Board provided ethical approval for the research (ref: 00041349).

### Participants and recruitment

A purposeful sampling strategy was used to recruit key stakeholders for interviews, including: LTC residents, care partners, and health professionals. Health professionals were any persons employed in Ontario’s health care system whose responsibilities included supporting residents and families through care transitions between the LTC and community care sectors. Residents were eligible to participate if they could communicate verbally in English, currently or previously resided in a LTC setting in Ontario, and experienced mild or no cognitive impairment. Care partners were family members or friends who provided support for a person who resided in a LTC setting in Ontario during the pandemic. All participants were required to have considered, supported, or experienced a care transition out of a LTC setting in Ontario during the COVID-19 pandemic. To achieve a balanced understanding of the factors influencing decision-making we aimed to recruit some participants who chose to transition and others who did not. This allowed us to consider the complexity of these decisions in our analyses. Multiple health organizations from across Ontario supported participant recruitment for this research by sharing study information within their networks. Relevant health organizations (e.g., Ontario Caregiver Organization, Ontario LTC Clinicians, Ontario Health) with an interest in aging, caregiving or the LTC and community care sectors were approached by the study team and a recruitment plan was developed for each consenting organization based on their desired level of involvement and available capacity. Depending on the organization, recruitment activities included emailing internal and external members, posting on social media, or distributing study information in a newsletter. A few participants were identified through snowball sampling, after learning about the study from a peer. Recruitment for this study was discontinued when sufficient information power was achieved [39].

### Data collection

Data were collected virtually through one-on-one semi-structured interviews. The research team developed an interview guide that sought to explore participants’ involvement in transition planning and the factors that guided their decision-making (Table 1). Participants were asked to describe who was involved in transition planning as well as their roles, whether the COVID-19 pandemic had influenced their transition decisions, and the factors that guided their decision-making. A trained doctoral candidate on the research team (SC) was responsible for data collection with the supervision and mentorship of senior researchers (KK, SL, WB). The research team had no relationship with participants prior to the study. Interviews were conducted by phone or videoconference (i.e., Zoom) depending on the participants’ preferences and lasted between 30 and 160 minutes in length (50 minutes on average). Interviews were audio-recorded and transcribed verbatim for analysis. Each participant was assigned a unique alphanumeric code (e.g., P01) to protect their identity.

**Table 1:** Sample interview questions.

	Interview question
1.	Tell me about yourself and your background.
2.	What was living in long-term care like before the pandemic?
3.	What types of care or support did you receive?
4.	What was living in long-term care like at the start of the COVID-19 pandemic?
5.	What made you consider leaving long-term care?
6.	Where did you think you might like to go?
7.	What influenced your decision to stay in, or move from, long-term care?
8.	Did you talk with anyone else or receive any support in making your decision?
9.	To what extent do you feel you were involved in care transition planning?
10.	Would you say your decision was relatively easy or hard? Why?
11.	How is everything going for you now?
12.	Do you have any advice for others who are considering leaving long-term care during the pandemic?

### Data analysis

Data were analyzed inductively and deductively using a content analysis approach [40]. Preliminary analyses were conducted concurrently with data collection to ensure sufficient information power was achieved [39]. Analytic memos were recorded by the interviewer (SC) immediately following each interview to identify potential points of interest, observed trends, and emerging questions [41]. These memos allowed the research team to establish greater familiarity with the content of the interviews and to adapt the interview guides to capture any new topics of interest. The interviewer (SC) and a senior researcher (KK) met frequently throughout data collection to discuss the content of the interviews. Once data collection was complete and interviews were transcribed, content analysis was used to organize and code the data [42]. Prior to this research, our team had conducted a scoping review to identify the key factors that stakeholders consider when making a transition decision [37]. As noted in the introduction section of this paper, seven key factors were identified: (a) institutional priorities and requirements; (b) resources; (c) knowledge; (d) risk; (e) group structure and dynamic; (f) health and support needs; and (g) personality, preferences and beliefs. These factors formed the initial codebook for this research and were refined to capture novel themes and insights. The final codebook was approved by the research team and one team member (SC) coded each transcript using NVIVO 12 software [43]. A senior researcher (KK) reviewed a selection of the coded transcripts to confirm agreement.

## Results

Thirty-two participants were interviewed from across Ontario. Table 2 provides a descriptive overview of participants’ characteristics. Participants were mostly care partners (n = 18), women (n = 21), over the age of 40 (n = 31) and self-identified as white (n = 28). Participants resided in different locations across Ontario, with the majority living in urban settings (n = 19). Most residents and care partners described experiences in licensed public or private LTC homes (n = 16).

**Table 2:** Demographic characteristics of participants (N = 32).

Characteristic	n (%)
Participant Type	
Resident	3 (9%)
Care partner	18 (56%)
Health Professional	11 (35%)
Sex	
Male	11 (34%)
Female	21 (66%)
Age group	
30–39	1 (3%)
40–49	8 (25%)
50–59	7 (22%)
60–69	9 (28%)
70–79	7 (22%)
Ethnicity	
White	28 (88%)
South Asian	2 (6%)
Persian	1 (3%)
Mixed heritage	1 (3%)
Geographic region in Ontario	
Urban	19 (59%)
Suburban	5 (16%)
Rural	8 (25%)
Care setting*	
Licensed LTC home	25 (78%)
High-needs retirement home	5 (16%)
Community care	2 (6%)
Resident moved out of LTC**	
Yes	10 (48%)
No	11 (52%)
Care partner-specific demographics (N = 18)	
Relationship to the resident	
Son/daughter	15 (83%)
Spouse/partner	2 (11%)
Friend	1 (6%)
Years of caregiving experience	
2 to 5 years	8 (44%)
> 5 years to 10 years	4 (22%)
> 10 years	5 (28%)
Not available	1 (6%)
Health professional-specific demographics (N = 11)	
Employment setting	
LTC setting	9 (82%)
Community setting	2 (18%)
Professional role	
Nursing	3 (27%)
Medicine	3 (27%)
Management/Administration (with clinical background)	5 (45%)
Years of experience	
5 to 10 years	1 (9%)
> 10 years	10 (91%)

*Some health professionals worked across multiple care settings (e.g., LTC and retirement home) and in this table we describe their primary employment setting.

**Sample does not include the health professional participants.

The sample included a range of different transition decisions. Of the residents and care partners, nearly half had completed a transition to a family home in the community (n = 10). A small portion of this group reported that the resident eventually moved back to their original LTC setting (n = 3). The remainder of resident and care partner participants carefully considered a potential care transition and decided against it for a variety of reasons (n = 11). Health professional participants had been involved in supporting decisions to both stay in, and move out of LTC, and thus spoke of the factors that were considered in either scenario.

Participants considered some or all of the seven factors that were identified in our scoping review when making their transition decisions: (a) personality, preferences, and beliefs; (b) health and support needs; (c) knowledge; (d) risk; (e) group structure and dynamic; (f) resources; and (g) institutional policies and procedures. Although these factors surfaced, in some form, among all three participant groups, the perceived importance of each factor was nuanced between groups. Participants’ extent of involvement in care transition decision-making was also varied between the groups interviewed. Although all participants generally considered each factor in their decision-making, health professionals tended to focus more heavily on risk and health and support needs, while residents and care partners had a larger focus on preferences and resources. Below, we describe who was involved in care transition planning, to what extent, and the factors that guided their decision-making in more depth. Table 3 provides a brief description of each factor with exemplary participant quotes. Although each of the factors are presented separately, there was some overlap between them which we acknowledge in the following sections.

**Table 3:** Description of decision-making factors related to LTC transitions, with exemplary quotes.

Factor	Description	Sample quotes
Personality, preferences and beliefs	The resident and care partners’ preferences for where the resident would live, their emotional responses to the pandemic, and their personal beliefs about caregiving.	“So I thought this is a better, more independent, more dignified choice for them. And I tried to put myself in their shoes, and ask ‘what would they want’” (CP 08) “This will never replace home sweet home” (R 29) “I think it was fear from COVID and the unknown” (P 20) “I talked to [resident] about it the week before. I said, ‘why don’t you come with me?’ And because I figured what was going to happen was that they were going to have to start locking them down. And so she wasn’t quite ready, and then we talked about it a week later and I really didn’t, I didn’t give her a lot of choice it was strongly encouraged…and of course I mean she was fine, she was totally fine to come when I explained what I thought I knew was going to come” (CP 12)
Health and support needs	The care needs of the resident and which environment could best meet these needs.	“In order for them to be eligible to be in a home is because they have needs…in order for them to go back into the community we are cognizant that the patients’ needs will not go away.” (P 33) “Well basically, this probably is the best place for me to get the necessary physical help. The medical attention [in LTC] is far superior” (R 27) “I think [LTC] was the safest place for him…all of his needs were met” (CP 03)
Knowledge	The individuals’ comfort in providing care, navigating the health system and how they processed emerging information on the pandemic context.	“I think that having worked in the field of aging, and aging in place, and healthy aging, it all came together to provide me with the tools to be able to support my mother and my own healthy aging” (CP 01) “It was like once we heard [journalist], and because of the conviction that he spoke with…there was no doubt that we would [move resident].” (CP 01) “I have the knowledge, I have the clinical knowledge that if anything went sideways, I could provide some support and care.” (CP 12)
Risk	The risks associated with staying in LTC or moving out.	“I desperately tried to get her out. I was so afraid that I had reason to worry about her health.” (CP 09) “I think the biggest [factor] was safety. ‘Is my loved one safe in this environment anymore?’” (P 02) “We were good in that this home was very careful” (R 27)
Group structure	The level of involvement of different stakeholders and the quality of their relationships.	“If everyone’s on the same page, wonderful. But as soon as one is not watch out” (CP 03) “Well during that month, I really advocated with my family, my siblings to get [resident] out. And that was a big big hoopla as well because they didn’t want [resident] to leave” (CP 15) “Let’s provide a lot of counselling to them, and then at the end of the day, if they still want to leave we want to make sure that wherever their home is we open up a home care referral for them…” (P 33) “Most of the residents were not able to [make the decision], some of them were, but I would say yes there was a lot of discussion with the substitute decision makers” (P 14)
Resources	The various tangible or intangible supports needed to transition successfully.	“Money is another aspect of that, that’s also a barrier, because you can only afford to do so much, you know, not everybody can afford to do everything.” (CP 09) “I think in a long-term care facility, you can’t make do with the staff that you have, you know there’s just not enough staff.” (CP 15) “So I think it would be a mixture of personal resources, being able to do that. The physical resources to have enough hands…and then the environmental resources to have them in your home.” (P 19) “It was always a financial hurdle or roadblock.” (R 13) “There’s a tool that from comes from the community, I think it’s called the Community ethics network. And there’s an ethical decision-making framework that I used that with where I did it sort of as a personal mental exercise, and then I shared what I would I thought of, with my family, so that we could kind of reflect on whether or not you know, we were making an ethical decision as a family. So that was helpful. I felt like we covered the domains of sort of ethical care and responsibility, and it was a thorough way to look at the problem and, and come up with a decision that we could live with.” (CP 28)
Policies and procedures	The impact of various policies on life in LTC and whether policies were in place to allow a transition.	“And so its like, where do you draw the line between keeping people safe and having a soft of quality of living…I’m really torn on that…when COVID hit its like ‘you’re not letting me in to see him, that’s not acceptable, I’m going to bring them home’” (CP 08) “I mean, you can equate it to somebody just being in jail, basically.” (P 21)

R = Resident; CP = Care Partner; HP = Health Professional

### Stakeholder involvement in decision-making

In most cases, care partners were the core decision-makers involved in planning a care transition in LTC during the pandemic. Their role was often focused on gathering information, advocating on behalf of the resident, and ensuring that appropriate resources were in place to facilitate a move. Participants from all three groups acknowledged that care partners often drove the decision to move a resident: “So I do know a couple of cases, especially when the pandemic started, where families took their loved ones home because they were terrified” (HP 32). When making their decisions, care partners sometimes spoke with health professionals; however, they appeared more likely to seek support from other resources. For example, participants commonly described connecting with various health and social care organizations (e.g., Alzheimer’s societies) and support groups to facilitate their decision-making. They also engaged their broader families in the decisions, and in particular their spouses who might be equally impacted by a care transition: “of course my husband was a participant in the decision-making because…I would not have forced him to accept my mother living with us” (CP 01). Oftentimes, there was significant tension and disagreement between care partners and their siblings regarding a potential transition for their parent. In one case a care partner even described hiring a lawyer to advocate to move their parent, since there was disagreement within their family.

Residents generally had limited involvement in transition decision-making. In most of the cases in this study, residents had some form of cognitive impairment and were believed to be unable to participate in decision-making, leaving their care partner responsible: “Most of the residents were not able to [participate], some of them were. But I would say yes there was a lot of discussion with the substitute decision-makers” (HP 14). Care partners approached resident involvement in decision-making in multiple ways. While many made an effort to involve the resident in decision-making, some care partners acknowledged that they might offer a tailored set of options to the resident, or might overrule the residents’ decision: “I talked to her about it the week before…and she wasn’t quite ready [to move]. And then we talked about a week later and I really didn’t give her a lot of choice, it was strongly encouraged” (CP 12). Of the three residents interviewed in the study, none of whom had cognitive impairment, all expressed that while decisions included them, they felt that they had little control over the decision outcomes.

Health professionals’ involvement in transition decision-making was highly varied. Health professionals employed in the LTC settings described their role as that of a care counsellor, helping care partners and residents to think through the practicality of their decisions: “I would tell them the pros and cons of both sides, and the things that they would need in the community” (HP 20). Health professionals in the LTC settings generally had a more intimate knowledge of the resident and so their perspective was viewed as important by community health professionals to ensure a safe transition. Health professionals employed in home and community care also played a critical role in bridging the transition from LTC to the home and supported decision-making by assessing the residents’ care needs and arranging home care services. Despite their importance to enabling a safe transition, home care staff were sometimes not informed of a transition in a timely fashion. This presented a challenge, as home care services were limited during the pandemic and sometimes took time to arrange: “I’ll give them a true picture of what to expect when they go out…[if] we are able to secure that right away or not” (HP 16). Overall, inclusion of both the LTC and home care perspectives were viewed as important but sometimes overlooked: “It’s certainly a missed opportunity for like a planned discharge meeting with and whether the long-term care home needed to be, like involved, but I think certainly Home and Community Care and some other community supports” (HP 31).

### Personality, preferences and beliefs

Participants’ decisions were often rooted in their personal beliefs, preferences, and emotional responses to the pandemic. In general, care partners wanted the resident to move out of LTC. Resident perspectives were more mixed, with some residents wanting to move while others wanted to stay: “I wanted to move out…this will never replace home sweet home” (R29). “I do know some where the family spoke to the loved one by phone and Facetime [who said] ‘what no, no I’m okay, leave me here’” (HP 32). Some care partners expressed that their decisions to move the resident were influenced by their personal beliefs about caring for an aging loved one: “We’re just trying to be true to our personal values, and we really, we don’t understand why more people don’t do it” (CP 17). In these cases, care partners often expressed how they viewed caring for a resident as a personal responsibility. Participants’ emotional reactions to the pandemic also influenced their decisions. Many participants described how their initial desire to pursue a transition was driven by fear: “[the decision] was fear motivated” (HP 31); or by guilt: “sometimes it’s that sense of obligation or guilt that comes in as a huge decision-making factor for someone as well” (CP 12). Finally, care partners also made transition decisions to avoid potential negative emotions in the future, like guilt or regret over not moving a resident when they had the ability to do so.

### Health and support needs

All stakeholders carefully considered where the residents’ health and support needs could best be met prior to making their decisions. Residents often had a combination of physical and mental health needs that required multiple types of support. In some cases, participants felt confident that these needs could be met in LTC during the pandemic: “I was not tormented by it, because he was in a quality place” (CP 03). Others believed that moving the resident would improve their quality of life. However, care partners and health professionals acknowledged that the residents’ care needs were extensive and would not disappear upon discharge: “by the very nature of being in long-term care, it means that person actually needs total care” (HP 02). Oftentimes, the residents’ health statuses had also worsened since their original admission to LTC, which meant that care partners would need greater support to care for them safely in the community than was needed when they first entered LTC. Unfortunately, many health professionals felt that care partners often did not recognize the full extent of the residents’ needs prior to making a transition decision: “…families completely underestimated how much care that person needed” (HP 32).

### Knowledge

Participants sought various forms of knowledge and information when making their decisions. The pandemic circumstances brought about significant uncertainty, and participants were required to process rapidly evolving information. Participants identified several types of knowledge that were necessary to their decision-making: knowledge of the resident and their care needs, knowledge of the health system, and knowledge of the pandemic. Having these types of knowledge helped to increase participants’ confidence in their decisions. As one care partner described: “I’ve seen [the resident] almost every day for three years…I could think it through with a complete set of information and not be second guessing” (CP 08).

Health professionals tried to fill gaps in residents’ and care partners’ knowledge, sharing information on the realities of a transition. As one health professional described: “just sort of having them go through, calm down, you can do this if you want, these are the things you should think about” (HP 02). Residents and care partners recalled seeking information from a variety of other sources as well, including trusted colleagues, friends, and organizations. The media also appeared to have a particularly influential role in guiding participants’ decision-making. Participants from all groups described how the media’s pandemic coverage drove them to consider a care transition: “It was like once we heard [journalist], and because of the conviction that he spoke with…there was no doubt that we would [move the resident]” (CP 1). In many cases, the negative portrayal of LTC in the media elevated participants’ feelings of fear and uncertainty, creating a sense of urgency to move the resident.

### Risk

When making their decisions, participants described weighing the risks of a resident staying in LTC, versus the risks of them moving into the community. Risks were typically centered around the residents’ safety and wellbeing. Care partners expressed concern that living in LTC would elevate the residents’ risk of contracting COVID-19. They were also deeply troubled by the idea that residents might be isolated to their rooms for extended periods of time due to the public health measures implemented in LTC settings during the pandemic. One care partner summarized these concerns: “So the fact that they were confined to their rooms, and that the risk of contagion was so great were the main reasons that I brought her to live with us” (CP 01). Some care partners and health professionals were also concerned about possible neglect in the LTC settings since they had already been struggling to supply adequate staffing prior to the pandemic. Participants acknowledged that transitioning the resident to the community could also result in new risks. Participants from all stakeholder groups generally perceived that risk of exposure to COVID-19 was lower in the community. However, they recognized that care transitions were often challenging for the resident who would need to adjust to a new environment. Care transitions also presented new risks for the care partners, who might burn out due to the decision: “if the caregiver is burnt out, they’re no good to that individual anyways” (CP 12). Finally, participants from all three groups were cognizant that limited availability of home care services meant that residents might have insufficient care while in the community.

### Group structure and dynamic

The quality and nature of the relationships between stakeholder groups also influenced decision-making. In general, participants sought to minimize friction and tension through their decisions. Care partners noted how pursuing a care transition might alter the nature of their relationship with the resident: “yeah so kind of the relationship that you have with your parent, and then that shifting where like, they changed your diapers at one point and now you’re changing their diapers” (CP 25). While some were comfortable with this shift, others were not. For example, one care partner described discomfort at the idea of providing physical care to their father: “I’m certainly not going to see my father naked, it’s just not how my relationship was. It would scar me” (CP 17). In some cases, residents and care partners had difficult relationships to begin with, making a transition feel less possible. One care partner recalled how the resident’s personality changed with the onset of dementia and how this impacted their decision: “I couldn’t manage the verbal abuse” (CP 28). The quality of the relationship between residents, care partners and health professionals was another important consideration. As one health professional described: “the ones that took the family home didn’t have a lot of trust in the [LTC] home to begin with” (HP 32).

Group structure also had an important influence on the transition decision-making process. As noted earlier, care partners were often the core stakeholder involved in decision-making. Although care partners endeavoured to solicit the input of residents and their broader families’ on their plans, they also articulated that the decision was theirs to make since they had been given power of attorney. This authority allowed them to navigate conflict and overrule decisions when required. As one care partner described: “My sister was not at all supportive, but I have power of care…I had full authority to make the decision” (CP 01). Although consulted on the transition plans, residents appeared comfortable allowing care partners to make the decisions, showing a high degree of trust. From participant accounts, decisions were often made through informal conversations rather than structured meetings. As a result, decision-making often appeared to be more individually-driven than group-driven, while many decisions did in fact engage multiple stakeholders.

### Resources

Resources were often a critical factor influencing transition decisions. Participants described a myriad of resources required to enable a safe transition, including: time, money, caregiving support, professional support, equipment, community services, and an accessible home to transition to. One health professional summarized the resources that they believed would be required: “I think it would be a mixture of personal resources, being able to do that. The physical resources to have enough hands in there… and then the environmental resources to have them in your home and accommodate wheelchair lifts and all that type of stuff” (HP 19). Participants who were able to facilitate a transition typically had many resources at their disposal. As one care partner described: “So we had the space, I had the knowledge and skills, and I had the family support to do it, so it was a bit of a no brainer” (CP 12). For many others, a lack of resources in one or more of these areas prevented a transition from occurring. For example, one care partner described how their decision was limited by the physical environment: “I frantically looked for homes…see, I had considered bringing her back to our place but we have stairs and we only have the one washroom on the second floor” (CP 09). Similarly, participants anticipated a lack of professional home care services while in the community: “I think it’s a barrier for people…for our residents if they were wanting to go home, there isn’t a lot of PSW support. They’re short-staffed, there’s a waiting list. And I think even just the whole system of how you can only get X number of hours a day, whilst if you get two hours a day from a PSW that still means a family member has to care for that person 22 hours a day” (HP 26). This gap in services is particularly important to acknowledge in transition planning due to how health care services are structured in Ontario LTC settings. Specifically, in Ontario LTC residents are assigned to the roster of a general practitioner affiliated with the setting, thus upon moving out the resident would lose their main point of access to the primary care system. As one care partner described: “What if I can’t get home care for mom, they won’t have a doctor, if they leave their facility that was the biggest thing, they won’t have a doctor” (CP 08). Therefore, despite a general interest and desire among residents and care partners to pursue a care transition, it was often viewed as unfeasible.

A small number of participants described using decision aids as a resource to facilitate the decision-making process itself. These tools outlined specific factors that residents and care partners should consider prior to pursuing a care transition. Some of the decision aids that were mentioned included the Ottawa Hospital’s COVID-19 LTC transition decision aid and the Community Ethics Network’s ethical decision-making worksheet. The few participants who accessed these tools felt that they helped them to think through the decision: “the tool was helpful I guess in reaffirming our decision. I might not have thought about my own doctor…so somewhere in there it became a very big thing that we had to have that or else this wasn’t going to work, so the tool was a helpful check on us, you know, a more objective check” (CP 06). Surprisingly, few health professionals described using these tools to support planning for a potential care transition. However, one health professional noted the importance of these tools moving forward: “I can anticipate having similar conversations with people [in the future] not with the same urgency, but exploring the same sort of framework that’s outlined in the tool about you know the ability to provide care and what it’s going to mean for a caregiver financially, psychologically, physically” (HP 14).

### Institutional policies and procedures

The institutional policies and procedures implemented in LTC settings were an important consideration during transition decision-making. As previously noted, isolation policies whereby residents were locked in their rooms to prevent the spread of COVID-19 motivated participants to move residents out of LTC. Many participants recognized the merits of these policies for protecting the resident against COVID-19 infection; however, some residents and care partners felt that the policies violated their rights: “it’s a human rights violation…I still to this day cannot believe that family was prevented from seeing their loved ones” (CP 09). Therefore, some care partners decided to move the resident to maintain access and ensure they could continue providing care.

Transition policies in LTC settings were another important consideration. During the pandemic, transition policies were amended to allow residents to move out of the homes for a period of up to 3-months and then return to the top of the waitlist for LTC re-admission. Although this change appeared supportive of care transitions, care partners were often leery of returning to a LTC waitlist. Some health professionals also suggested that residents and care partners would be giving up their bed if they moved. Despite this, some health professionals described flexibility: “so we actually took her back, which was nice of us because we didn’t have to” (HP 11).

## Discussion

This research explored Ontarians’ decisions to stay in, or move from, LTC settings during the COVID-19 pandemic. Specifically, this paper describes who was involved in the decisions, to what extent, and the factors that guided their decision-making. Our findings stand to inform future transition policies and procedures in LTC settings, through insights on how individuals approached transition decision-making during the pandemic. To accomplish this, 32 participants (a mix of residents, care partners, and health professionals) were interviewed from across Ontario. The findings show that transition decisions were largely driven by care partners, with some input from residents, health professionals and other trusted stakeholders. The seven transition decision-making factors identified in our team’s recent scoping review [37] featured prominently in the interviews. These factors include: (a) personality, preferences and beliefs; (b) health and support needs; (c) knowledge; (d) risk; (e) group dynamics; (f) resources; and (g) institutional policies and procedures. Thinking through these factors allowed participants to determine whether a care transition was necessary, desirable, and feasible. For many participants, it was a complex decision that required alignment between the different factors to become possible.

There were some notable differences in how the participant groups considered and evaluated each factor during decision-making. Differences were most apparent between care partners and health professionals and appeared most pronounced for the following factors: personality, preferences, and beliefs; knowledge; and risk. When assessing risk, for example, care partners often focused more heavily on the risks of remaining in LTC, whereas health professionals were more concerned with the risks associated with moving. As a result, they often held differing perspectives on whether a transition was warranted. Earlier research has shown that stakeholders may hold differing perspectives and goals during care transitions, which can produce conflict [44, 45]. While some conflict between stakeholders was observed in this study, differing opinions between stakeholders often created tension in transition planning and may have contributed to a less collaborative and communicative approach. Thus, recognizing that stakeholders may hold different perspectives increases the importance of engaging them in the planning process so that their views can be incorporated into decisions.

The multiple factors that were identified as influential of a transition from LTC to community reveal how privilege and inequity shape health decision-making. Health researchers have previously noted how it ‘takes a village’ to transition a patient along the continuum of care [46]. Consistent with this, our research showed how LTC to community transitions required multiple enabling factors, including: supportive policies and procedures, knowledge of the resident and health care system, availability of caregiving support, strong relationships between stakeholders, access to medical care in the community, financial resources, and an appropriate physical environment. Residents and care partners who had these factors in place had increased agency and decision-making power enabling them to consider and pursue a care transition. For those without these enabling factors, a transition to the community was unfeasible due to a lack of supports and resources in place. Our research captured both perspectives, yet it is important to recognize that a third group exists: those who lacked sufficient resources and supports to even consider a transition. At its core, decision-making involves choosing between different actions and options [47]. Thus, by only recruiting participants who considered a transition our research overlooked those who did not even believe transitioning was an option. Further research should seek to explore the perspectives of these groups who were likely among the most vulnerable people in LTC.

Transition decisions were undoubtedly shaped by the evolving crisis of the COVID-19 pandemic. Prior to the pandemic, it was estimated that less than 1% of LTC residents would transition from LTC to community care [48, 49]. During the pandemic stakeholders faced unbridled uncertainty [50] and suffered indirect health, social and economic hardships [51]. These crisis circumstances appear to have shifted stakeholders’ decision framing [52], with participants in this study stating that if not for the pandemic they never would have considered a transition. Crisis Decision Theory (CDT) may offer a useful lens to understand this apparent shift in decision-making. Specifically, CDT connects theories of stress and coping to decision research to understand and predict decision responses to negative events [53]. It focuses on the decision processes that occur in a crisis and the factors that predict peoples’ choices [53]. For example, CDT theorists have emphasized the important role of emotion in crisis decision-making suggesting that emotions can allow one to respond more quickly, easily, and efficiently to uncertainty and that different types of emotions may contribute to differing responses [54]. In this study, emotions appeared to play a more dominant role in decision-making than in other types of transitions, with participants describing how intense feelings of guilt and fear drove care partners’ desires to leave LTC. Although beyond the scope of this paper, further research may explore whether different emotional responses contributed to different transition decisions and pathways and how emotional responses can be better recognized and supported in transition planning. Similarly, CDT suggests that people may process information differently when making decisions in crisis situations [55]. During a pandemic, news media plays a crucial role in communicating information to the public and can also influence decision-making [56]. Participants in our study discussed how pandemic media coverage amplified the perceived risks associated with LTC and contributed to a sense of urgency to transition for care partners. Most commonly, they referred to media pieces on the most severe COVID-19 outbreaks, and where trusted sources or care partners shared their perspectives on LTC in the pandemic. Prior research has shown the persuasive effect of narrative information on crisis decisions [55]. Government and healthcare institutions may benefit from exploring this effect in greater depth so that they can better understand how public responses are influenced by the media.

### Practical implications

This research highlighted important gaps in Ontario’s healthcare systems which threaten the safety of LTC to community transitions. In our interviews, participants described limited home care services, and a poor transfer of professional accountability across care settings. Oftentimes, care partners are expected to fill these gaps in care; however, this can lead to negative outcomes for both residents and care partners [57]. Transitions were further challenged by the fact that most residents in Ontario do not retain their general practitioner upon LTC admission [58], and thus would lose access to their assigned primary care provider if they moved out of LTC. Together, these concerns depict a fragmented care system where residents’ care options are limited, and, particularly in times of crises, can easily fall through the cracks. The Government of Ontario has previously recognized structural issues in the healthcare system that have contributed to fragmentation, poor coordination and uneven distribution of care across regions [59]. Unfortunately, many of these concerns have been exacerbated by the COVID-19 pandemic, where the home care sector in particular has faced a significant staffing crisis [60]. Although steps have been taken to reduce the gaps in Ontario’s healthcare systems, fresh attention should be paid to ensuring continuity of care between LTC and home care, as this type of transition is relatively new, has less established processes in place, and is likely to occur again in a future pandemic.

This research also offers important insight into the roles of each stakeholder group during transition planning. For this study we interviewed residents, care partners and health professionals because we believed that each group would likely be involved in transition decision-making. Our interviews showed that transition decisions were largely made by care partners, sometimes in consultation with residents and health professionals. This represents a notable change in decision-making roles, as prior research has found that health professionals typically lead transition decision-making in institutional care settings [37]. This shift may be a result of the many consequences of the COVID-19 pandemic in LTC settings, which produced considerable trauma among care partners [61] and may have prompted them to take action. Alternatively, it may be the result of resource shortages which limited health professional capacity to support decision-making processes. In this study, health professionals tended to focus more heavily on certain factors when supporting care partners and residents with transition planning. Specifically, health professionals commented on the potential risks involved with a transition, the health and support needs of the resident, and the resources that would be required to safely care for a resident in the community. Although health professionals should generally recognize the importance of all seven factors in transition planning, it makes sense that these factors were emphasized given the speed at which decisions were being made and professionals’ desires to protect the residents and care partners.

Consistent with other studies, resident involvement in transition decision-making was often limited [37, 62]. Participants in the study noted cognitive impairment as justification for their limited resident involvement; however, persons with advanced cognitive impairment may still be able to show understanding and indicate a choice with appropriate support [63]. In fact, residents in this study expressed mixed preferences about staying in LTC, yet their preference was not always realized. Other researchers have recognized the complexities of involving older adults in health decision-making and identified a series of supportive practices for stronger engagement, including focusing decision-making on patient and family values and ensuring interprofessional partnerships founded on effective communication [64]. Future research might helpfully explore the value of these practices in care transitions as explicit consideration of resident perspectives and preferences may contribute to transition success and alleviate decision-making burden on other stakeholder groups.

Health professionals’ role in transition decision-making was largely focused on information sharing and counselling. In this study, we spoke with a variety of health professionals who were involved in LTC transitions, either in the LTC settings themselves or in the community. In general, health professionals worked to guide decision-making by ensuring that the resident and family were adequately prepared and informed. Multiple decision aids have emerged during the pandemic to support transition decision-making in LTC [18, 65]. These tools provide relevant information on transition policies and procedures, encourage individuals to reflect on the rationale behind their decision, and identify factors that may enable or challenge their care transition. Another key benefit of these tools is that they encourage active participation in decision-making [66]. Despite this, few of the participants in our study knew that these tools existed. Moving forward, health professionals may find these tools particularly useful as they seek to guide residents and care partners through transition planning.

Additionally, although both LTC and community health professionals played an important role in supporting care transitions, there were often inadequate processes in place to ensure strong communication and coordination of care. Community health professionals described situations where they had not been alerted to the fact that a resident was discharged to the community until it was too late to safely organize home care services. This was particularly challenging in the pandemic, when staffing shortages meant that home care services were more difficult to access. Prior research has highlighted the importance of engaging home and community health professionals in transition planning to support continuity of care and the implementation of discharge plans [67]. Ensuring that both LTC and community health professionals are involved in transition planning and decision-making early on can help to reduce these risks and support greater continuity in care.

### Limitations

This research aimed to provide a descriptive understanding of decisions to move out of LTC settings in Ontario during the COVID-19 pandemic. Although we feel this study contributed substantively to the existing evidence and understanding of this area of decision-making, there are some important limitations to this work. Notably, due to the pandemic circumstances, recruiting diverse participant perspectives to this work was a challenge. For example, residents in LTC settings often have poor access to phones or computers in their rooms which limited their participation, and many residents had advanced forms of cognitive impairment and were not eligible to participate. To ensure that resident perspectives were included in this research, we engaged a broad number of care partners to act as a proxy. In the interview, we asked care partners to describe the residents’ perspectives to the best of their knowledge. Further, in cases where a residents’ eligibility was uncertain, we attempted to connect with a care partner who acted as their surrogate decision maker. Although we anticipated that transition decisions may have been impacted by an individual’s background and culture, we also had difficulty recruiting participants from equity deserving groups. To support the inclusion of equity deserving groups, we reached out directly to various health organizations in Ontario that provided support to these populations, but this was met with limited success. Equity deserving groups face unique barriers to research participation and are often underrepresented in research. However, their inclusion is important to offer greater depth in understanding, make results more transferable and avoid perpetuating existing inequalities [68]. Equity deserving groups also face longer wait times to enter ethnic-specific LTC [69] and have also been disproportionately impacted by the COVID-19 pandemic [70], which may have influenced their transition decisions. Future research should set out to consider the perspectives of these groups to ensure a more comprehensive understanding of transition decision-making. Finally, in this study we did not collect information about contextual aspects of specific LTC settings that our participants transitioned from, even though transition decisions may have been heavily influenced by the severity of COVID-19 outbreaks in these settings. We chose not to collect this information to protect the privacy of our participants, due to the substantial media coverage around certain LTC settings during the pandemic. Yet, greater detail on the outbreaks experienced in these settings would have allowed us to explore how people responded to different severities of crises.

## Conclusions

This study explores decisions to stay in, or move out of, LTC settings from the perspectives of residents, care partners and health professionals in Ontario. The findings afford important insights towards who was involved in the planning process and the factors that influenced stakeholders’ transition decisions during the COVID-19 pandemic. This knowledge is important for care providers and policy-makers to consider moving forward, as transitions like these are likely to continue unless the conditions in LTC settings are improved. Further research should focus on how residents, care partners and health professionals can be better supported to make these decisions in the future. These findings can also be used to strengthen the development of decision aids and other tools to support decision-making in a public health crisis.

## Supporting information

S1 Checklist COREQ (COnsolidated criteria for REporting Qualitative research) checklist. (PDF)
